# Oral Problems in Brazilian Individuals with Rare Genetic Diseases That Affect Skeletal Development

**DOI:** 10.3390/ijerph21091227

**Published:** 2024-09-18

**Authors:** Flavia Rabello, Mariana Laís Silva Celestino, Natália Cristina Ruy Carneiro, Jennifer Reis-Oliveira, Heloísa Vieira Prado, Mauro Henrique Nogueira Guimarães de Abreu, Ana Cristina Borges-Oliveira

**Affiliations:** 1 Undergraduate Course in Dentistry, Pontifical Catholic University of Minas Gerais, Belo Horizonte 30535-500, Brazil; flaviarabello@pucminas.br; 2 Department of Social and Preventive Dentistry, School of Dentistry, Federal University of Minas Gerais, Belo Horizonte 31270-800, Brazil; mlscelestino@ufmg.br (M.L.S.C.); jenniferreisoliveira@ufmg.br (J.R.-O.); heloisaprado@ufmg.br (H.V.P.); maurohenrique@ufmg.br (M.H.N.G.d.A.); 3 Department of Pediatric Dentistry, School of Dentistry, Federal University of Minas Gerais, Belo Horizonte 31270-800, Brazil; nataliacrcarneiro@ufmg.br

**Keywords:** dental care for disabled individuals, mucopolysaccharidosis, osteogenesis imperfecta, rare diseases

## Abstract

The present study aimed to compare the prevalence of oral problems between individuals with rare genetic diseases that affect skeletal development and individuals without rare diseases. A cross-sectional study was conducted with 210 individuals between two and fifty-four years of age: 105 with rare genetic diseases (27 with mucopolysaccharidosis [MPS] and 78 with osteogenesis imperfecta [OI]) and 105 without rare diseases. The rare genetic disease group was recruited from hospital units that provide care for patients with MPS and OI in five states of Brazil, and the other group was recruited from the same location. The participants were examined with regards to malocclusion, dental anomalies, dental caries, and gingivitis. A questionnaire was administered addressing individual, sociodemographic, and behavioral characteristics as well as dental history. A descriptive analysis was performed, followed by unadjusted and adjusted binary logistic regression analyses. The mean age was 14.1 ± 12.2 years. Individuals with a rare disease were 12.9-fold more likely to have some type of oral problem (95% CI: 3.7–44.7) compared to the group without rare diseases. The prevalence of oral problems was higher among Brazilians with MPS and OI compared to normotypical individuals.

## 1. Introduction

A disease is defined as rare when its prevalence is less than 65 cases per 100,000 people [1]. Individually, the prevalence of a rare disease is low, but when grouped with other rare diseases, the epidemiological impact is quite substantial and assumes the proportion of a public health problem [1,2,3,4,5,6]. An estimated 5000 to 8000 rare diseases affect 3.5 to 5.9% of the world population, corresponding to 400 million individuals [4,5,6]. In Brazil, an estimated 13 million individuals are diagnosed with some type of rare disease [7].

Rare diseases are generally chronic, degenerative, progressive, and disabling, requiring continual multidisciplinary care [2,3,6,8]. Most are of a genetic origin (80.0%) and hereditary in many cases [1,2,4,5]. Signs and symptoms generally differ among diseases and can even vary among individuals with the same genetic anomaly [3,6,8,9,10,11,12]. Approximately 15% of rare diseases have oral and maxillofacial manifestations [13].

One of the impairments of a rare disease is the abnormal development of bone and joint structures, which is present in more than 400 types of diseases. Such abnormalities may be found in several regions of the body, including facial bones and structures [8,11,14,15]. Mucopolysaccharidosis (MPS) and osteogenesis imperfecta (OI) are two rare genetic diseases in which affected individuals have dentofacial abnormalities [11,12,14,15,16,17,18]. Abnormalities related to craniofacial growth and dental anomalies are common in individuals with rare genetic diseases that affect skeletal development and can predispose such individuals to occlusal problems. An abnormal position of the teeth in the arch and changes in bone structure and tooth formation, together with a cariogenic diet and deficient brushing, can serve as predisposing factors for dental caries and gingivitis [11,12,14,15,17]. Susceptibility to dental caries does not depend only on individual aspects, but also on collective and contextual factors [11,14,17,18].

Dentofacial abnormalities can increase the prevalence of oral problems, compromising aspects such as breathing, chewing, speech, and esthetics. Malocclusions, dental anomalies, dental caries, and periodontal disease are among the oral problems found in affected individuals. According to the literature, individuals diagnosed with MPS and OI are more prone to oral problems due to physical and motor abnormalities, which can increase the difficulty in performing proper oral hygiene. This is also due to the fact that such individuals have greater difficulty with regards to access to dental care [8,9,10,11,12,14,18,19,20]. 

The identification of the most prevalent oral problems in individuals with rare genetic diseases compared to the general population is of considerable importance to the development of public policies, the formulation of health promotion, and intervention strategies directed at oral health care [8,9,10,11,12,14,17]. Thus, the present study aimed to compare the prevalence of oral problems between Brazilian individuals with rare genetic diseases that affect skeletal development and Brazilian individuals without rare diseases. The hypothesis is that the prevalence of oral problems is higher among individuals with rare conditions compared to normotypical individuals.

## 2. Materials and Methods

The study was approved by the Human Research Ethics Committee of the Federal University of Minas Gerais (certificate numbers: 01480212.4.0000.5149 for MPS and 4755516.4.0000.5149 for OI).

A cross-sectional study was conducted with 210 individuals between two and fifty-four years of age: 105 with rare genetic diseases that affect skeletal development [MPS (*n* = 27) and OI (*n* = 78)] and 105 without rare diseases. The groups were named G1 and G2, respectively. G1 was recruited from medical clinics of reference services and care units specialized in MPS and OI. Data collection took place in five Brazilian states (Ceará, Espírito Santo, Minas Gerais, Rio de Janeiro, and São Paulo). Individuals without rare diseases were recruited from other clinics of the same hospitals. A convenience sample was used due to the rare characteristic of the diseases investigated and the low overall prevalence, which made the use of sample size estimations quite challenging. The snowball sampling recruitment method was employed to increase the number of participants [21]. The study was conducted between January and December 2019. To be included in G2, the participants could not have any rare disease or any other disability or clinical/sensorial abnormality (physical/intellectual disability, syndromes, autism spectrum disorder, chronic/acute diseases, progressive or neurodegenerative conditions). The two groups were matched for sex and age at a proportion of 1:1.

### 2.1. Data Collection

A clinical oral examination was performed in both groups, and a questionnaire addressing sociodemographic characteristics and dental history was administered in interview format to individuals 18 years of age or older without intellectual impairment as well as to the parents/guardians of minors. This questionnaire was adapted from the instruments used by Prado et al. (2019) [11].

MPS and OI types were collected through the patients’ medical charts. Skin color was recorded following the criteria established by the Brazilian Institute of Geography and Statistics [22]. Family income was determined by the total monthly income of all economically active members of the family using the monthly minimum wage as the reference (October 2022: USD 217.00).

The intraoral exams were conducted in a medical care room. The entire process was carried out with the use of an artificial light (Petzl Zoom head lamp, Petzl America, Clearfield, UT, USA), mouth mirror (PRISMA^®^, São Paulo, SP, Brazil), and Community Periodontal Index (CPI) probe (Golgran^®^, São Paulo, SP, Brazil). The participant was positioned in a conventional chair, wheelchair, or hospital bed. The examiner used personal protective equipment. No radiographic exams were performed.

The outcome was “presence of oral problems”. Malocclusion, dental anomalies, dental caries, and gingivitis were the conditions assessed clinically. Individuals with at least one of these conditions were classified in the category “oral problem present”. 

Participants were diagnosed with malocclusion according to the presence/absence of the following conditions: overjet (increased/protrusion, anterior crossbite, absent), overbite (deep bite, anterior open bite, absent, upper), and posterior crossbite [11,12,14,22]. Dental anomalies were recorded [conoid tooth, agenesia, microdontia, rotated teeth, development defects of enamel (DDEs), and dentinogenesis imperfecta (DI)]. As only a clinical examination was performed, agenesia was considered a possible diagnosis [11,12]. DDEs were recorded using the DDE index [23,24]. DI was determined based on Petersen and Wetzel (1998) and Abukabbos and Al-Sineedi (2003) [16,25]. Dental caries was diagnosed using the criteria recommended by the WHO. The number of teeth with cavitated lesions was recorded for the primary and permanent dentitions [11,12,14,21]. Gingivitis was investigated by performing an analysis of the contour and color of the gingival tissue [12,26].

### 2.2. Training and Calibration Process

Three previously trained examiners performed the oral examinations. The training and calibration process comprised two steps. The first step consisted of theoretical training, including the analysis of images of the clinical conditions investigated. The second step comprised the calibration process, which occurred on two occasions with a 10-day interval involving five adolescents with MPS or OI and five adolescents without rare conditions at previously selected locations (state of Minas Gerais). Intra-examiner Kappa coefficients ranged from 0.78 to 1.00 (1.00 for malocclusion, 0.97 for dental anomaly, 0.96 for dental caries, and 0.78 for gingivitis). Inter-examiner Kappa coefficients ranged from 0.77 to 1.00 (1.00 for malocclusion, 0.93 for dental anomaly, 0.91 for dental caries, and 0.77 for gingivitis).

### 2.3. Pilot Study

This step was performed after the calibration process and involved five individuals with MPS or OI and five individuals without rare diseases and their parents/guardians at one of the locations previously selected for the study (state of Minas Gerais). As the results revealed no need to alter the proposed methods, the participants of this phase were included in the final sample.

### 2.4. Directed Acyclic Graph

**Figure 1:**
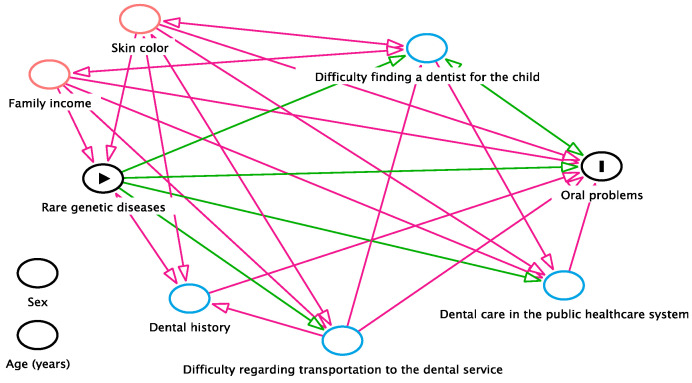
Directed acyclic graph (DAG) and possible confounding variables in the associations between rare diseases and oral problems.

A directed acyclic graph (DAG) was used as a visual method of representation of causal presuppositions. This method selects covariables for statistical adjustment and identifies confounding factors for the causal relationship in question [11,27]. To identify possible confounding variables, the authors included some sociodemographic characteristics, dental history, and clinical conditions in the study (skin color, family income, dental history, dental care in the public healthcare system, difficulty finding a dentist for the child, difficulty regarding transportation to the dental service, and oral problems). As the participants in the two groups were matched for age and sex, these variables were not incorporated into the DAG. Based on the described theoretical model, skin color and family income were identified as confounders of the association between rare genetic conditions and oral problems (Figure 1).

### 2.5. Statistical Analysis

The Statistical Package for the Social Sciences (SPSS for Windows, version 26.0, IBM Inc., Amonk, NY, USA) was used to conduct all statistical analyses. Descriptive statistics were performed for the data. Binary logistic regression models (unadjusted and adjusted) were performed to estimate odds ratios (ORs) using the conditional backward method, considering a 95% confidence interval (CI) and individual matching for age and sex. 

Logistic regression models depend on the estimates of their coefficients. Multicollinearity can cause problems in the adjustment of the model and exert an impact on the estimates of the parameters. Thus, the variance inflation factor (VIF) was used to measure collinearity. Cook’s distance was used to assess influential cases. Lastly, the Hosmer–Lemeshow test was used to measure the correspondence between predicted and observed values of the dependent variable, considering a *p*-value < 0.05. The power of the study was measured with the G*Power software, version 3.1.9.4, considering the final sample, a 95% confidence interval, and the proportion of discordant pairs for the outcome (oral problems) between paired cases and controls.

## 3. Results

The mean age of the 210 participants was 14.1 ± 12.2 years (median: 9.5 years) and the female sex predominated (*n* = 113; 53.8%). In G1 (one hundred and five individuals), twenty-seven participants had a diagnosis of MPS, eight of whom had type I (29.7%), five had type II (18.5%), three had type III (11.1%), two had type IV (7.4%), and nine had type VI (33.3%). Among the 78 individuals with OI, 37 had type I (47.4%), 22 had type III (28.3%), 4 had type IV (5.1%), and 11 had no defined type (14.1%). The type of OI was not recorded on the charts of four patients (5.1%).

Table 1 shows the sociodemographic characteristics, dental history, and clinical conditions in G1 and G2. The oral problems identified during the clinical examinations are displayed in Table 2.

“Rare genetic disease”, “skin color”, and “family income” remained in the final model. The VIF for these variables was close to 1 (1.094 for rare disease, 1.084 for skin color, and 1.016 for family income). Therefore, collinearity was not a problem in the regression model. Cook’s distances were lower than 1, indicating no influential cases in the model. Goodness of fit was considered adequate, as demonstrated by the Hosmer–Lemeshow test (result: 0.099). The only association was between “rare disease” and “oral problems” (Table 3). G1 participants (with rare disease) were 12.9-fold more likely to have some oral problem (95% CI: 3.7–44.7) than G2 participants (normotypical).

Considering a 95% confidence interval, an unadjusted OR of 12.4, a total sample of 105 individuals, and a 25.7% proportion of discordant pairs, the power of the two-tailed a posteriori test was 99.4%.

## 4. Discussion

The present study compared the presence of oral problems between individuals with MPS or OI and normotypical individuals. Due to the difficulty in performing oral hygiene, many individuals with rare genetic diseases require assistance from another person, even adolescents and adults. However, parents and caregivers often perform this task inadequately due to a lack of information or fail to perform it, increasing the susceptibility to gingival problems and dental caries [8,11,12,19,20]. A study conducted in Brazil involving 70 individuals with rare genetic diseases that affect skeletal development (MPS and OI) and 70 individuals without rare diseases showed that the rare disease group was more vulnerable to dental caries [11]. According to the authors, individuals with inadequate oral hygiene and a rare condition have a greater likelihood of being diagnosed with dental caries. Moreover, it is important to point out that individuals with OI exhibit ultrastructural dentinal changes due to collagen defects [28]. Thus, dentists should be aware of this, as such individuals are more prone to developing dental caries.

Malocclusion and dental anomalies were the most prevalent oral problems among the participants in the present study. The high frequency of malocclusion in G1 participants may be explained by the presence of skeletal dysplasia, which alters bone development of the skull and face. A study carried out by Koehne et al. (2018) showed that the prevalence of open bite, distal occlusion, and increased overjet is higher in patients with MPS due to condyle resorption [17]. Arriagada-Vargas et al. showed that tooth agenesis, microdontia, and developmental defects of enamel were the most prevalent abnormalities among participants with rare diseases compared to normotypical individuals. The authors also found high rates of overbite, maxillary hypoplasia, mouth breathing, labial interposition, and cleft lip/palate [15].

The high prevalence of oral problems among G1 participants in the present study underscores the need for dental care targeting this population. However, a lack of knowledge on the particularities of rare diseases among the majority of dentists and the lack of experience in providing care for such patients make dental professionals feel insecure and unprepared to offer them care. This situation hinders the access of patients diagnosed with rare genetic diseases to dental care in both the public and private sectors, leaving this portion of the population even more susceptible to oral problems [11,12,14,18,19,20]. 

Dental professionals qualified in providing care for patients with rare genetic conditions are fundamental to the practice of high-quality dental services. In a study conducted in Germany, Hanisch et al. (2018) showed that 20.0% of parents of individuals with rare conditions had difficulties finding a dentist capable of providing care for their child. The parents revealed that many dentists do not have adequate knowledge or experience in the treatment of rare diseases and referred the patients to dental specialty centers [10].

In the present sample, no associations were found with dental history, difficulty finding a dentist, or difficulty regarding transportation to the dental service. However, the participants were recruited from care centers or reference units for rare diseases, and therefore, the majority already had access to treatment and care through the public healthcare system, including referrals for dental care.

Despite the existence of a universal healthcare system in Brazil, studies have shown that difficulty in gaining access to dental services is an obstacle that can affect oral health care for individuals with disabilities in the country. Individuals with disabilities generally do not receive the same benefits or dental care offered to the general population, making them more susceptible to oral problems [11,12,14,20].

Complex care management for individuals with rare diseases, frequent medical appointments, therapies, and hospitalizations makes parents place more importance on medical treatments and not pay sufficient attention to oral health status [11,12,14,18,20]. As many rare genetic diseases do not have a cure, follow-up by the multidisciplinary team enables these individuals and their families to receive counseling on fundamental care [3,6,11,14]. In this context of multidisciplinary care, it is essential for the team that provides care for these individuals to have knowledge on the orofacial abnormalities and oral problems often found in patients with rare genetic diseases. Adequate information provided to parents and caregivers on oral health, including counseling on the need for preventive dental care, can make all the difference in the quality of life of individuals with rare diseases.

Often, a late diagnosis, a lack of knowledge on the diseases, and the scarcity of qualified healthcare providers and services are part of the experience of the majority of individuals with rare genetic diseases and their families [2,3,5,6,11,13,14,18,29]. A study developed by Iriart et al. (2009) found that the therapeutic itineraries of this population in the search for diagnoses and care are influenced by social context [3].

No significant association was found between oral problems and skin color or family income in the present study. The literature shows that Black, Brown, and Indigenous individuals in Brazil are more likely to experience socioeconomic vulnerabilities, which can have repercussions on the lives of the families of individuals with rare diseases and increase the difficulty in gaining access to qualified healthcare providers and services [11,14,26]. Social vulnerability is a frequent finding among individuals with rare diseases in Brazil. A study conducted by Pinto et al. (2019) showed that Brazilian families of individuals with rare diseases experience a significant economic burden in terms of family income [26]. The literature reports that the costs of dental treatment are high, which underscores the importance of preventive care for patients with rare diseases [11,12,14,20].

Some limitations of the present study should be taken into account. A questionnaire was used for data collection, and recall bias is a possibility. The cross-sectional design impedes the establishment of causal inferences. Considering the rare characteristics of the group studied, a convenience sample was chosen. However, the authors selected the participants at reference centers responsible for the care of these patients. Moreover, the authors used the snowball sampling method to expand the sample as much as possible. The use of a comparison group and the DAG tool to identify possible confounding variables minimized influences on the association between rare genetic diseases and oral problems.

It is of the utmost importance for healthcare professionals who provide care for individuals with rare diseases to have a better understanding and knowledge of the oral health status of these individuals or to at least consider the importance of oral health care [6]. According to the authors cited, to improve knowledge on genetic diseases, oral health should have a prominent place in the phenotyping of patients. Therefore, interdisciplinary care involving healthcare providers from different fields is fundamental.

Identifying the prevalence of oral problems and associated factors in this population enables in-depth knowledge that can help healthcare providers improve dental care and offer better counseling for caregivers. This can contribute to the elaboration of adequate public health policies. Individuals with rare genetic diseases constitute a minority in the general population and do not have the same visibility as other population groups. Public policies that ensure the fundamental right to health, integral care, and access to qualified healthcare providers and services are fundamental to attenuating the susceptibility to oral problems in this portion of the population.

## 5. Conclusions

The prevalence of oral problems was higher among G1 participants (with rare genetic diseases) compared to G2 participants (normotypical). Malocclusion and dental anomalies were the most prevalent oral problems among the participants with rare diseases. No associations were found with dental history, difficulty finding a dentist, or difficulty regarding transportation to the dental service.

## Figures and Tables

**Table 1:** Sample distribution according to sociodemographic characteristics, dental history, and clinical condition (*n* = 210).

Sample Characteristics	Genetic Condition		
	With Rare Disease (G1) *n* (%)	Without Rare Disease (G2) *n* (%)	Total *n* (100.0%)
State			
Ceará	7 (50.0)	7 (50.0)	14
Espírito Santo	36 (64.3)	20 (35.7)	56
Minas Gerais	42 (42.9)	56 (57.1)	98
São Paulo	10 (45.5)	12 (54.5)	22
Rio de Janeiro	10 (50.0)	10 (50.0)	20
Skin color			
White	31 (33.7)	61 (66.3)	92
Not white (Black/Brown/Yellow)	74 (62.7)	44 (37.3)	118
Family income ^a^			
≥monthly minimum wage ^b^	99 (52.1)	91 (47.9)	190
<monthly minimum wage	4 (28.6)	10 (71.4)	14
Dental history			
Yes	95 (49.0)	99 (51.0)	194
No	10 (62.5)	6 (37.5)	16
Dental care (public health system)			
Yes	83 (60.6)	54 (39.4)	137
No	22 (30.1)	51 (69.9)	73
Difficulty finding dentist for child ^c^			
Yes	50 (78.1)	14 (21.9)	64
No	45 (34.6)	85 (65.4)	130
Difficulty with transportation to dental service ^c^			
Yes	25 (86.2)	4 (13.8)	29
No	70 (42.4)	95 (57.6)	165
Oral problems ^d^			
Absent	3 (9.7)	28 (90.3)	31
Present	102 (57.0)	77 (43.0)	179

^a^ Missing values/^b^ Brazilian monthly minimum wage (October 2022) = USD 217.00; ^c^ registration of 194 participants with dental history; ^d^ malocclusion, dental anomalies, dental caries, and gingivitis.

**Table 2:** Sample distribution according to oral problems (*n* = 210).

Oral Problems	Genetic Condition		
	With Rare Disease (G1) *n* (%)	Without Rare Disease (G2) *n* (%)	Total *n*
Malocclusion			
Absent	14 (24.1)	44 (75.9)	58
Present	91 (59.9)	61 (40.1)	152
Dental anomalies			
Absent	18 (21.2)	67 (78.8)	85
Present	87 (69.6)	38 (30.4)	125
Dental caries			
Absent	71 (43.8)	91 (56.2)	162
Present	34 (70.8)	14 (29.2)	48
Gingivitis			
Absent	76 (44.4)	95 (55.6)	171
Present	29 (74.4)	10 (25.6)	39

**Table 3:** Factors associated with oral problems in G1 and G2 participants (*n* = 210).

Variable	Percentage of Oral Problems (%)	Unadjusted Odds Ratio (95% CI)	*p*-Value	Adjusted Odds Ratio (95% CI)	*p*-Value
Rare disease					
Absent (*n* = 105)	97.1	1	<0.001	1	<0.001
Present (*n* = 105)	73.3	12.4 (3.6–42.2)		12.9 (3.7–44.7)	
Skin color					
White (*n* = 92)	80.4	1	0.087	1	0.538
Not white (*n* = 118)	89.0	2.0 (0.9–4.3)		0.8 (0.3–1.8)	
Family income ^a^					
≥monthly min. wage ^b^ (*n* = 190)	84.2	1	0.399	1	0.185
<monthly min. wage (*n* = 14)	92.9	2.4 (0.3–19.3)		4.2 (0.5–34.8)	

CI—confidence interval; ^a^ missing values/^b^ Brazilian monthly minimum wage (October 2022) = USD 217.00.

## Data Availability

The data that support the findings of this study are available upon request from the corresponding author. The data are not publicly available due to restrictions, e.g., because they contain information that compromises the privacy of the research participants. All listed authors meet the authorship criteria and all authors agree with the content of the manuscript.
